# Comparison of software vs. cognitive‐based fusion‐targeted biopsies for prostate cancer diagnosis

**DOI:** 10.1002/bco2.70248

**Published:** 2026-07-12

**Authors:** Marcus Westerberg, Rolf Gedeborg, Hans Garmo, Fredrik Jäderling, Pär Stattin, David Robinson

**Affiliations:** ^1^ Department of Surgical Sciences Uppsala University Uppsala Sweden; ^2^ Department of Molecular Medicine and Surgery (MMK) Karolinska Institutet Stockholm Sweden; ^3^ Department of Radiology Capio S:t Görans Hospital Stockholm Sweden; ^4^ Department of Urology Skåne University Hospital Malmö Sweden; ^5^ Department of Urology Höglandssjukhuset Eksjö Sweden

**Keywords:** cognitive‐based fusion biopsy, magnetic resonance imaging, prostate cancer, software‐based fusion biopsy, targeted biopsy

## Abstract

**Objectives:**

This study aimed to compare the effectiveness of software‐based fusion‐targeted biopsies (STBx) versus cognitive fusion‐targeted biopsies (CogTBx) in detecting prostate cancer (PCa) and clinically significant PCa.

**Materials and methods:**

Men aged 30–85 were included in a target trial emulation if they had undergone a STBx or CogTBx of Prostate Imaging Reporting and Data System (PI‐RADS) 3–5 lesions in 2020–2024. Using log‐link binary regression models with inverse probability of treatment weighting to adjust for confounding, we estimated the associations between biopsy technique and detection of PCa and clinically significant PCa (Gleason ≥3 + 4 and Gleason ≥4 + 3) by use of adjusted relative risks (aRR) with 95% confidence intervals (CIs) obtained via bootstrapping.

**Results:**

A total of 2092 men who underwent STBx and 7933 men who underwent CogTBx in 2020–2024 were identified in PCBase Xtend. The overall proportion diagnosed with PCa was 64%. STBx detected PCa with a slightly higher frequency (aRR, 1.05; 95% CI, 1.02–1.09), corresponding to an absolute increase of 3.3 percentage points (95% CI 1.2–5.3). The relative risk for detection of Gleason ≥4 + 3 was somewhat larger (aRR, 1.13; 95% CI, 1.03–1.23). The largest relative difference for detection of PCa was observed in PI‐RADS 3 lesions (aRR, 1.12; 95% CI, 1.01–1.24), and this pattern became even more apparent when the outcome was restricted to clinically significant PCa.

**Conclusions:**

Software‐based fusion‐targeted biopsies detected slightly more PCa and clinically significant PCa compared to cognitive fusion‐targeted biopsies, particularly in men with PI‐RADS 3 lesions. Prioritizing PI‐RADS 3 lesions for software‐based targeted fusion biopsy could optimize diagnostic effectiveness.

## 1 BACKGROUND

The introduction of magnetic resonance imaging (MRI) in the diagnostic work‐up of men with elevated prostate‐specific antigen (PSA) has reduced the number of men who undergo biopsy.^1^ When MRI indicates a lesion with a Prostate Imaging Reporting and Data System (PI‐RADS) score of 3–5, the lesion can be sampled using software‐based fusion‐targeted biopsies (STBx), cognitive‐targeted biopsies (CogTBx) or in‐bore biopsies. The latter is rarely used in current practice. In randomized controlled trials, MRI‐targeted biopsies of the prostate in men with elevated PSA detected more Gleason score ≥7 prostate cancer (PCa) than systematic biopsies and less Gleason 6 cancers.^2^, ^3^

STBx integrate the lesion detected by MRI with the ultrasound image. For a CogTBx the operator visually combines the location of the lesion on the MRI with the ultrasound image. This technique is therefore more operator dependent. In a meta‐analysis of 20 studies with a total of 4928 men who had undergone STBx or CogTBx there was no difference in PCa detection between the two techniques.^4^ A direct randomized comparison of these biopsy techniques is unlikely to be performed, so a comparative effectiveness study based on a target trial emulation would be of value.^5^

The aim of this study was to compare STBx and CogTBx in a target trial emulation and estimate the incidence of any PCa and of clinically significant prostate cancer according to two definitions (Gleason score ≥3 + 4 and Gleason ≥4 + 3) in men with PI‐RADS 3–5 lesions. We also aimed to evaluate this in subgroups according to PI‐RADS and age.

## 2 MATERIAL AND METHODS

### 2.1 Data sources

The National Prostate Cancer Register (NPCR) of Sweden collects data on PCa care to assess adherence to national guidelines. It has a coverage of >98% of all PCa diagnoses compared to the Cancer Register to which registration is mandated by law.^6^ In Prostate Cancer data Base Sweden (PCBase), NPCR has been linked to other healthcare registers and demographic databases, including the Cancer Register, the Patient Register, the Prescribed Drug Register and the Total Population Register. In PCBase Xtend (eXtended Treatments and ENDpoints), longitudinal data on PSA, prostate biopsies and MRI has been extracted from healthcare IT systems in all Swedish regions.^7^

Data on type and date of prostate biopsy (procedure codes KEB [any form of prostate biopsy], TKE10 [CogTBx, transrectal], TKE13 [CogTBx, transperineal], TKE20 [STBx, transrectal], TKE23 [STBx, transperineal]) and the centre (i.e., hospital or clinic), where the biopsy was performed, were extracted from the Patient Register. The most common used systems in Sweden were bkFusion with MIM Software (GE HealthCare), Koelis fusion systems (Koelis) and ARTEMIS Prostate Fusion Biopsy System (InnoMedicus Artemis). PI‐RADS and prostate volume were extracted from the MRI reports using a rule‐based text recognition algorithm.^8^ Longitudinal PSA data were obtained directly from laboratory information systems. Date of PCa diagnosis, Gleason score, use of systematic and targeted biopsies, T stage, M stage and primary treatment were obtained from NPCR. We also complemented NPCR with data from the Cancer Register (date of diagnosis, T and M stage) and data on Gleason score for targeted biopsies in pathology reports from laboratory information systems.

Use of 5‐alpha reductase inhibitors (Anatomical Therapeutic Chemical codes G04CB01, G04CB02) was extracted from the Prescribed Drug Register. Health‐adjusted life expectancy was calculated based on age and comorbidity at date of biopsy.^9^

The Swedish Research Ethics authority approved the study and waived the requirement for informed consent.

### 2.2 Study design

We designed a target trial emulation with the intention to compare biopsy technique (STBx and CogTBx) as intervention. The target trial and the operational definitions for the intervention are described in detail in Table S1.^10^

### 2.3 Study population

Men aged 30–85 were included if they had undergone a STBx or CogTBx of the prostate in 2020–2024. We excluded men with a previous PCa diagnosis, men who had undergone prostate biopsy performed within 2 years before the biopsy, and men who had been on 5‐alpha reductase inhibitors during 2 years preceding the biopsy.^11^ Men were also excluded if they resided in a region where data on PSA and MRI was not available at the date of biopsy, if the most recent PSA before the biopsy was above 20 ng/mL, if there was no MRI finding categorized as PI‐RADS 3–5 performed within 6 months before the biopsy, or if prostate volume was not possible to extract from the MRI report. Men with multiple biopsy dates were included at the date of their first biopsy.

Some centres started to perform transperineal biopsies in 2023–2024, shortly after the introduction of transrectal fusion biopsies. Since there are indications that transperineal biopsies detect more PCa than transrectal biopsies,^12^ we excluded centres that during a particular calendar year performed >10% of the biopsies using the transperineal approach. The main study population covered biopsies performed at 63 different centres.

### 2.4 Exposure

Information on type and date of prostate biopsy and the centre where the procedure was performed was extracted from the Patient Register. Type of biopsy was categorized based on procedure code as (a) STBx/transrectal [TKE20], CogTBx/transrectal [TKE10], STBx/transperineal [TKE23] or CogTBx/transperineal [TKE13]. The procedure code for any form of prostate biopsy [KEB] was in addition used to exclude men with a previous biopsy.

### 2.5 Outcomes

A biopsy was considered resulting in a PCa diagnosis if the diagnosis was registered in NPCR or the Cancer Register within 30 days from the biopsy. Outcomes were the diagnosis of any PCa without consideration of Gleason score and the diagnosis of clinically significant PCa according to two definitions (Gleason ≥3 + 4 and Gleason ≥4 + 3).

### 2.6 Statistical analysis

We estimated the relative risk (RR) and absolute risk difference (RD) for STBx using CogTBx as reference in binary regression models with log link. These estimates were adjusted for age at biopsy, year of biopsy, PSA, prostate volume and PI‐RADS. Adjustment was made using inverse probability of treatment weighting (IPTW) where the propensity score for treatment (i.e., biopsy technique) was modelled with logistic regression. We used overlap weights that handles nonoverlapping propensity score distributions and achieve exact balance of means of covariates included in the IPTW model.^13^ In particular this means that the standardized mean differences of variables included in the IPTW model are equal to zero.

In the analyses of clinically significant PCa as outcome, men with missing Gleason score at diagnosis were excluded. We also conducted subgroup analyses based on PI‐RADS and age (<70 years and ≥70 years).

Since the main analysis population included both transrectal and transperineal biopsies but excluded biopsies performed at centres with >10% transperineal biopsies, we performed two sensitivity analyses. In the first analysis, all biopsies from all centres were included, irrespective of the proportion transperineal biopsies performed, and in the other analysis, only transrectal biopsies were included. We also performed a conservative sensitivity analysis where missing Gleason scores were set to 6 in men who underwent STBx and to 8–10 in men who underwent CogTBx.

We estimated 95% confidence intervals using percentile‐based bootstrap (1000 resamplings). The statistical analyses were performed using R 4.0.2.

## 3 RESULTS

### 3.1 Baseline characteristics

**FIGURE 1:**
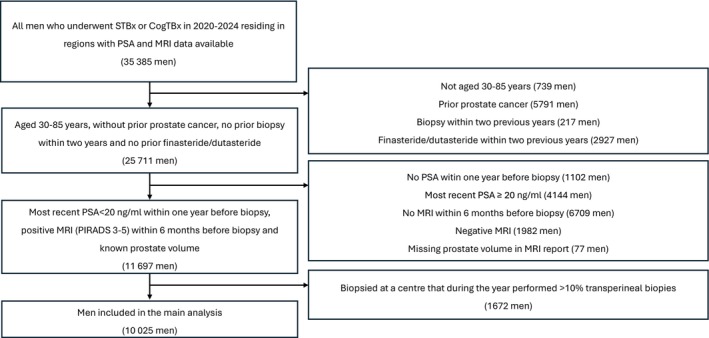
Study flow chart. CogTBx, cognitive fusion‐targeted biopsies; STBx, software‐based fusion‐targeted biopsies.

The baseline characteristics of the 10 025 men in the main study population were similar among men who underwent STBx (*N* = 2092) and CogTBx (*N* = 7933), in terms of age, clinical cancer characteristics and life expectancy (Table 1 and Figure 1).

**TABLE 1:** Baseline characteristics of 10 025 men who underwent software‐based fusion‐targeted biopsies (STBx) versus cognitive fusion‐targeted biopsies (CogTBx).

	STBx		CogTBx	
	*N*	(%)	*N*	(%)
*N*	2092	(100)	7933	(100)
Type of biopsy				
Transperineal	46	(2)	41	(1)
Transrectal	2046	(98)	7892	(99)
Age at biopsy (years)				
Median (IQR)	67 (62–73)		68 (62–74)	
30–59	385	(18)	1492	(19)
60–69	959	(46)	3339	(42)
70–79	701	(34)	2792	(35)
80–85	47	(2)	310	(4)
Year of biopsy				
2020–2021	1464	(70)	4537	(57)
2022–2024	628	(30)	3396	(43)
PI‐RADS				
3	595	(28)	2264	(29)
4	939	(45)	3161	(40)
5	558	(27)	2508	(32)
Volume (cm^3^)				
Median (IQR)	44 (33–60)		43 (33–58)	
<50	1260	(60)	4933	(62)
≥50	832	(40)	3000	(38)
PSA (ng/ml)				
Median (IQR)	6.0 (4.3–8.6)		6.3 (4.5–9.1)	
<3.0	158	(8)	430	(5)
3.0–9.9	1590	(76)	5898	(74)
10.0–19.9	344	(16)	1605	(20)
PSA density (ng/mL/cm^3^)				
Median (IQR)	0.13 (0.09–0.19)		0.14 (0.10–0.21)	
<0.10	616	(29)	2059	(26)
0.10–0.19	1002	(48)	3674	(46)
≥0.20	474	(23)	2200	(28)
Health‐adjusted life expectancy (years)				
Median (IQR)	18 (14–22)		18 (13–22)	
<10	162	(8)	761	(10)
10–19	1154	(55)	4326	(55)
≥20	776	(37)	2846	(36)

Abbreviations: IQR, inter quartile range; PI‐RADS, Prostate Imaging Reporting and Data System; PSA, prostate‐specific antigen.

### 3.2 Use pattern of biopsy technique

Biopsy technique varied according to calendar year and centre (Figure S1). STBx was the dominating biopsy technique in 13% of centres in 2020 and this proportion increased to 25% in 2024. The majority of centres (52/71) had a low and stable proportion of STBx; some centres had a stable and high proportion of STBx (8/71), while some centres (11/71) appeared to gradually transition from CogTBx to STBx during the study period. Centres contributed with between one and 665 men to the main analysis, and the centre‐specific proportions of detected PCa were generally similar, and the detection was not clearly associated with the centre‐specific number of men (Figure S2).

In men diagnosed with PCa, STBx was less frequently (19%) combined with systematic biopsies, compared with CogTBx (40%) (Table 2). The transperineal approach was infrequent, with a use of 2% (46/2092) among men who underwent STBx and 0.5% (41/7933) among men who underwent a CogTBx (Table 1).

**TABLE 2:** Characteristics of men diagnosed with prostate cancer.

	STBx		CogTBx	
	*N*	(%)	*N*	(%)
***N***	1342	(100)	5049	(100)
Type of biopsy				
Transperineal	31	(2)	30	(1)
Transrectal	1311	(98)	5019	(99)
Age at biopsy (years)				
Median (IQR)	69 (64–74)		69 (63–75)	
30–59	187	(14)	782	(15)
60–69	592	(44)	1979	(39)
70–79	524	(39)	2022	(40)
80–85	39	(3)	266	(5)
Year of biopsy				
2020–2021	927	(69)	2746	(54)
2022–2024	415	(31)	2303	(46)
PI‐RADS				
3	256	(19)	939	(19)
4	608	(45)	2030	(40)
5	478	(36)	2080	(41)
Volume (cm^3^)				
Median (IQR)	40 (31–55)		40 (31–53)	
<50	918	(68)	3499	(69)
≥50	424	(32)	1550	(31)
PSA (ng/ml)				
Median (IQR)	6.3 (4.6–8.9)		6.8 (4.9–9.7)	
<3.0	72	(5)	180	(4)
3.0–9.9	1022	(76)	3694	(73)
10.0–19.9	248	(18)	1175	(23)
PSA density (ng/ml/cm^3^)				
Median (IQR)	0.15 (0.11–0.22)		0.16 (0.12–0.24)	
<0.1	283	(21)	881	(17)
0.1–0.19	662	(49)	2362	(47)
≥0.2	397	(30)	1806	(36)
Gleason				
6	395	(29)	1448	(29)
3 + 4	531	(40)	2035	(40)
4 + 3	235	(18)	698	(14)
8	98	(7)	417	(8)
9–10	79	(6)	432	(9)
Missing	4	(0)	19	(0)
T stage				
1–2	1234	(92)	4617	(91)
3–4	69	(5)	299	(6)
Missing	39	(3)	133	(3)
M stage M1				
Yes	28	(2)	80	(2)
Additional systematic biopsies*				
No	1085	(81)	3001	(59)
Yes	255	(19)	2029	(40)
Missing	2	(0)	19	(0)
Primary treatment*				
Active surveillance	383	(29)	1481	(29)
Radical treatment	845	(63)	2957	(59)
Watchful waiting or androgen deprivation therapy	107	(8)	434	(9)
Other/missing	7	(1)	177	(4)

Abbreviations: CogTBx, cognitive fusion‐targeted biopsies; STBx, software‐based fusion‐targeted biopsies.

* Data only available in NPCR and not the Cancer Register.

### 3.3 Association between biopsy technique and detection of PCa

PCa was detected in 64% after both STBx and CogTBx (Table 3). Most cancers were Gleason 6 (28%) or Gleason 3 + 4 (39%). In total, 23 men (<0.2%) with PCa had unknown Gleason, with similar proportions in the two groups (Table 2).

**TABLE 3:** Gleason score on biopsy according to PI‐RADS on MRI prostate and age. STBx = software‐based fusion‐targeted biopsies, CogTBx = cognitive fusion‐targeted biopsies.

Gleason score	Subgroup	STBx			CogTBx		
		*N*	*n*	(%)	*N*	*n*	(%)
Any	All	2092	1341	(64)	7933	5045	(64)
	PI‐RADS 3	595	256	(43)	2264	938	(41)
	PI‐RADS 4	939	607	(65)	3161	2027	(64)
	PI‐RADS 5	558	478	(86)	2508	2080	(83)
	Age 30–69 years	1344	778	(58)	4831	2758	(57)
	Age 70–85 years	748	563	(75)	3102	2287	(74)
≥3 + 4	All	2088	943	(45)	7914	3582	(45)
	PI‐RADS 3	594	144	(24)	2260	495	(22)
	PI‐RADS 4	938	424	(45)	3153	1372	(44)
	PI‐RADS 5	556	375	(67)	2501	1715	(69)
	Age 30–69 years	1340	505	(38)	4826	1778	(37)
	Age 70–85 years	748	438	(59)	3088	1804	(58)
≥4 + 3	All	2088	412	(20)	7914	1547	(20)
	PI‐RADS 3	594	48	(8)	2260	138	(6)
	PI‐RADS 4	938	170	(18)	3153	499	(16)
	PI‐RADS 5	556	194	(35)	2501	910	(36)
	Age 30–69 years	1340	192	(14)	4826	657	(14)
	Age 70–85 years	748	220	(29)	3088	890	(29)

Abbreviations: CogTBx, cognitive fusion‐targeted biopsies; STBx, software‐based fusion‐targeted biopsies.

**FIGURE 2:**
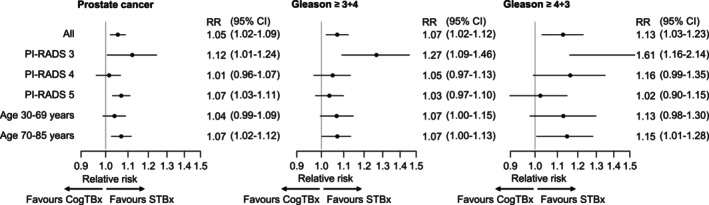
Adjusted relative risk (RR). Software‐based fusion‐targeted biopsies (STBx) versus cognitive fusion‐targeted biopsies (CogTBx). CI = confidence interval.

PCa was slightly more often detected with STBx than with CogTBx (adjusted RR, 1.05; 95% CI 1.02–1.09) (Figure 2). This corresponded to an adjusted absolute risk difference of 3.3 percentage points (95% CI 1.2–5.3 percentage points) and a number needed to treat of 30 (Figure S3).

Risks were similar when outcome was restricted to Gleason ≥3 + 4, (adjusted RR, 1.07; 95% CI 1.02–1.13) and an adjusted absolute risk difference of 3.0 percentage points (95% CI 0.9–5.2 percentage points) (Figure 2 and Figure S3). Further restricting the outcome definition to Gleason ≥4 + 3 slightly strengthened the association but with reduced precision (adjusted RR, 1.13; 95% CI 1.03–1.23), and the absolute risk difference was smaller (adjusted RD, 2.3 percentage points; 95% CI 0.5–4.0 percentage points).

### 3.4 Subgroup analyses

When stratified by PI‐RADS, the association between STBx and the risk for any PCa was stronger in men with PI‐RADS 3 (adjusted RR, 1.12; 95% CI 1.01–1.24) (Figure 2 and Figure S3). This pattern became even more apparent when the outcome was restricted to clinically significant PCa, and the association was strongest for the outcome Gleason ≥4 + 3 (adjusted RR, 1.61; 95% CI 1.16–2.14). The analyses stratified by age group did not indicate any notable heterogeneity.

### 3.5 Sensitivity analyses

The two sensitivity analyses of (i) all biopsies performed and (ii) only transrectal biopsies provided similar results as the main analysis (Figure S4). Results were also similar in the sensitivity analysis where missing Gleason values were imputed using a conservative approach, assigning a fixed worst case value to minimize the risk of overestimating a positive effect of STBx.

### 3.6 Assessment of propensity scores

Propensity score distributions overlapped between the two groups (Figure S5).

## 4 DISCUSSION

In this target trial emulation comparing STBx to CogTBx, PCa was somewhat more frequently detected in men who underwent STBx. The adjusted risk difference of 3.3 percentage points corresponds to a number needed to treat of 30 to detect one extra case of PCa. The relative risk for detection of clinically significant PCa (Gleason ≥4 + 3) was somewhat stronger, but the absolute risk difference was somewhat lower. The association between STBx biopsies and cancer detection appeared stronger in men with PI‐RADS 3 but was estimated with lower precision in this subgroup analysis.

Previous studies addressing this question have been hampered by methodological limitations and small sample size. A meta‐analysis of 20 previous studies, including a total of 4928 men, found no difference in the diagnostic performance of software‐based and cognitive fusion biopsy.^4^ The pooled estimates of detection of mainly Gleason ≥4 + 3 were 37% for cognitive and 39% for software‐based fusion (i.e., a relative risk of 1.05). In the analysis of Gleason ≥3 + 4, the pooled estimates were 36% (CI: 23%–50%) for cognitive and 35% (CI: 27%–43%) for software‐based fusion. Limitations of this meta‐analysis includes the small sample size, different study designs, inclusion criteria and definitions of clinically significant PCa, and detection rates, of the included studies. There are consequently limited and uncertain findings from previous studies.

A target trial emulation using population‐based data on PSA, MRI and biopsy results can potentially overcome some of the key limitations of previous studies. We believe that a randomized trial directly comparing the two biopsy techniques is unlikely to be performed and therefore our target trial emulation is of value.^5^ The validity for causal interpretation of any target trial emulation relies on the three assumptions of causal inference: positivity, exchangeability and consistency.^14^, ^15^

Positivity assumes that every individual has a nonzero chance of receiving any of the interventions. This assumption ensures that the treatment is not deterministic and that there is some randomness in the treatment assignment. We argue that the choice of biopsy technique mainly depended on healthcare provider preference, as indicated by the large variation between centres in the use of technique. There are no obvious patient characteristics that would disqualify a man from one of the biopsy techniques.

Exchangeability assumes that the groups compared are exchangeable, meaning that there are no other systematic differences between the groups with an impact on the outcome. In our case, comprehensive information on patient characteristics, including health‐adjusted life expectancy, PSA, prostate volume and PI‐RADS allowed for adjustment of key potential confounders. The IPTW approach for control of confounding is appropriate for unconditioned time‐fixed exposures and resulted in good overlap of propensity score distributions. This supports exchangeability between the comparison groups and limits the concern for potentially inadequate control of confounding.

The consistency assumption for causal inference means that the potential outcome of a particular intervention is the same as the observed outcome of that intervention. Translated into the context of the current research question: Would a man randomized to a biopsy technique, independent of surgeon, have the same outcome as the outcomes observed in our study? This question puts focus on the potential impact of operator performance. Higher operator experience may improve cancer detection rates.^16^, ^17^, ^18^ The majority of centres had a low proportion of STBx, some centres had a stable and high proportion, while there also were centres in the study that increased their use of STBx during the study period. Since most centres appear to have a stable preference, there is no obvious mechanism that would link operator skill to biopsy technique. We have therefore not identified any serious concern arising in relation to the consistency assumption. There may however be effect heterogeneity across centres that we were unable to account for due to the large variation in the number of men included in the study across centres and the dominance of one biopsy technique in many centres. We may therefore have underestimated the width of the CIs. There was however no indication of an association between the number of men and proportion of detected PCas, and the proportions were generally similar.

The study also relies on the validity of MRI findings and pathological diagnoses. Interobserver variability has been observed in PI‐RADS scoring and Gleason grading, but we do not expect the variability in our study to be differential according to biopsy technique.^19^, ^20^ MRI evaluation takes place before biopsy and misclassification should therefore be non‐differential and, if present, cause a conservative bias that attenuates the association towards the null. While the pathological examination is done after biopsy, no plausible mechanism for a differential misclassification has been identified, and misclassification would be expected to be conservative.

In men with a diagnosis of PCa, CogTBx were combined with systematic biopsies twice as often as for STBx. This information was not available for men not diagnosed with PCa. However, PCa diagnosis is made after the biopsy and can therefore not influence the selection of biopsy technique, so this difference in proportion undergoing also systematic biopsies is therefore expected to be representative for the entire study population. It is consequently unlikely that the tendency for increased cancer detection with STBx is related to concomitant systematic biopsies. The lower use of systematic biopsies can rather be seen as another potential advantage of the STBx technique.^2^, ^3^

We argue that our estimates are applicable to men that undergo targeted transrectal biopsies for PI‐RADS 3–5 with PSA below 20 ng/mL and that have not recently undergone a biopsy in similar settings. The reason for a marginally superior performance of software‐based fusion biopsy may be attributed to the fact that PI‐RADS 3 lesions are frequently not visualized on transrectal ultrasound, thereby complicating cognitive targeting.^21^ Our finding that STBx biopsies increased detection of clinically significant PCa in men with PI‐RADS 3 was estimated with lower precision but suggests that if the capacity for software‐based fusion biopsies is limited, giving priority to men with PI‐RADS 3 lesions could optimize diagnostic effectiveness.

## 5 CONCLUSIONS

This study suggests that software‐based fusion‐targeted biopsy may improve the detection of prostate cancer and clinically significant prostate cancer in routine practice, particularly for PI‐RADS 3 lesions, compared with cognitive fusion‐targeted biopsy.

## AUTHOR CONTRIBUTIONS

**Marcus Westerberg:** Writing—original draft preparation; writing—review and editing; conceptualization; data curation; formal analysis; investigation; methodology; software; validation; visualization. **Rolf Gedeborg:** Writing—review and editing; conceptualization; investigation; methodology. **Hans Garmo:** Writing—review and editing; conceptualization; data curation; investigation; software. **Fredrik Jäderling:** Writing—review and editing. **Pär Stattin:** Writing—review and editing; conceptualization; funding acquisition; investigation; resources; supervision. **David Robinson:** Writing—original draft preparation; writing—review and editing; conceptualization; funding acquisition; investigation; supervision.

## CONFLICT OF INTEREST STATEMENT

The authors declare no conflicts of interest.

## DISCLOSURE

Rolf Gedeborg is employed by the Medical Products Agency (MPA) in Sweden. The MPA is a Swedish Government Agency. The views expressed in this article may not represent the views of the MPA.

## Supporting information


**Table S1.** Target trial specification. STBx = Software‐based fusion‐targeted biopsies, CogTBx = cognitive fusion‐targeted biopsies.
**Figure S1.** Centre biopsy technique preference, in centres that contributed to the main analysis, among all men who underwent software‐based fusion‐targeted biopsies (STBx) and cognitive fusion‐targeted biopsies (CogTBx) at these centres in 2020–2024.
**Figure S2.** Number of men, biopsy technique and proportion with positive outcome according to centre in the main analysis. Centres were sorted according to the total number of men. STBx = Software‐based fusion‐targeted biopsies, CogTBx = cognitive fusion‐targeted biopsies.
**Figure S3.** Absolute risk difference in the main analysis. Software‐based fusion‐targeted biopsies (STBx) versus cognitive fusion‐targeted biopsies (CogTBx). AR = absolute risk difference, CI = confidence interval.
**Figure S4.** Relative risk and risk difference in the sensitivity analyses. Software‐based fusion‐targeted biopsies (STBx) versus cognitive fusion‐targeted biopsies (CogTBx). RR = relative risk. RD = absolute risk difference, CI = confidence interval.
**Figure S5.** Distribution of inverse probability of treatment weights in the main analysis. STBx = Software‐based fusion‐targeted biopsies; CogTBx = cognitive fusion‐targeted biopsies.

**Data S1.** Supporting Information.

## Data Availability

Data used in the present study was extracted from the Prostate Cancer Database Sweden (PCBase), which is based on the National Prostate Cancer Register (NPCR) of Sweden and linkage to several national health‐data registers. The data cannot be shared publicly because the individual‐level data contain potentially identifying and sensitive patient information and cannot be published due to legislation and ethical approval (https://etikprovningsmyndigheten.se). Use of the data from national health‐data registers is further restricted by the Swedish Board of Health and Welfare (https://www.socialstyrelsen.se/en/) and Statistics Sweden (https://www.scb.se/en/), which are Government Agencies providing access to the linked healthcare registers. The data will be shared on reasonable request in an application made to any of the steering groups of NPCR and PCBase (contact npcr@npcr.se or par.stattin@uu.se). To request data or analytic code from this study, contact the corresponding author. For detailed information, please see www.npcr.se/in-english, where registration forms, manuals, and annual reports from NPCR are available alongside a full list of publications from PCBase.

## References

[1] Robinson D , Abdulkareem R , Nasrollah D , Ljung A , Hintze P , Wallby S , et al. Frequency of biopsy and tumor grade before vs after introduction of prostate magnetic resonance imaging. JAMA Netw Open. 2023;6(8):e2330233. 10.1001/jamanetworkopen.2023.30233 37606924 PMC10445184

[2] Kasivisvanathan V , Rannikko AS , Borghi M , Panebianco V , Mynderse LA , Vaarala MH , et al. MRI‐targeted or standard biopsy for prostate‐cancer diagnosis. N Engl J Med. 2018;378(19):1767–1777. 10.1056/nejmoa1801993 29552975 PMC9084630

[3] Ahmed HU , El‐Shater Bosaily A , Brown LC , et al. Diagnostic accuracy of multi‐parametric MRI and TRUS biopsy in prostate cancer (PROMIS): a paired validating confirmatory study. Lancet. 2017;389:815–822. 10.1016/S0140-6736(16)32401-1 28110982

[4] Falagario UG , Pellegrino F , Fanelli A , Guzzi F , Bartoletti R , Cash H , et al. Prostate cancer detection and complications of MRI‐targeted prostate biopsy using cognitive registration, software‐assisted image fusion or in‐bore guidance: a systematic review and meta‐analysis of comparative studies. Prostate Cancer Prostatic Dis. 2025 Jun;28(2):270–279. 10.1038/s41391-024-00827-x 38580833 PMC12106061

[5] Hernán MA , Robins JM . Using big data to emulate a target trial when a randomized trial is not available. Am J Epidemiol. 2016;183(8):758–764. 10.1093/aje/kwv254 26994063 PMC4832051

[6] Tomic K , Berglund A , Robinson D , Hjälm‐Eriksson M , Carlsson S , Lambe M , et al. Capture rate and representativity of the National Prostate Cancer Register of Sweden. Acta Oncol. 2015;54(2):158–163. 10.3109/0284186X.2014.939299 25034349

[7] Westerberg M , Holm L , Garmo H , Stattin P , Gedeborg R . Cohort profile update: the National Prostate Cancer Register of Sweden and PCBase. Int J Epidemiol. 2025;54(6):dyaf172. 10.1093/ije/dyaf172 41078339 PMC12516312

[8] Ventimiglia E , Gedeborg R , Westerberg M , Zaurito P , Jäderling F , Stattin P , et al. Nationwide population‐based longitudinal data on magnetic resonance imaging of the prostate and subsequent prostate biopsy results. Scand J Urol. 2026;61:64–71. 10.2340/sju.v61.45540 41879676

[9] Westerberg M , Ahlberg M , Orrason AW , Gedeborg R . Assessment of variability in life expectancy in older men by use of new comorbidity indices. A nationwide population‐based study. Scand J Urol. 2024;59:207–209. 10.2340/sju.v59.42504 39704547

[10] Kutcher SA , Brophy JM , Banack HR , Kaufman JS , Samuel M . Emulating a randomised controlled trial with observational data: an introduction to the target trial framework. Can J Cardiol. 2021;37(9):1365–1377. 10.1016/j.cjca.2021.05.012 34090982

[11] Thompson IM , Goodman PJ , Tangen CM , Lucia MS , Miller GJ , Ford LG , et al. The influence of finasteride on the development of prostate cancer. N Engl J Med. 2003;349(3):215–224. 10.1056/NEJMoa030660 12824459

[12] Bryant RJ , Marian IR , Williams R , Lopez JF , Mercader C , Raslan M , et al. Local anaesthetic transperineal biopsy versus transrectal prostate biopsy in prostate cancer detection (TRANSLATE): a multicentre, randomised, controlled trial. Lancet Oncol. 2025 May;26(5):583–595. 10.1016/S1470-2045(25)00100-7 40139210

[13] Li F , Morgan KL , Zaslavsky AM . Balancing covariates via propensity score weighting. J am Stat Assoc. 2018;113(521):390–400. 10.1080/01621459.2016.1260466

[14] Smit J , Krijthe J , Kant W , Smit JM , Krijthe JH , Kant WMR , et al. Causal inference using observational intensive care unit data: a scoping review and recommendations for future practice. Npj Digit Med. 2023;6(1):221. 10.1038/s41746-023-00961-1 38012221 PMC10682453

[15] Rosenbaum PR , Rubin DB . The central role of the propensity score in observational studies for causal effects. Biometrika. 1983;70(1):41–55. 10.1093/biomet/70.1.41

[16] Leprêtre M , Chaoui I , Bruyère F , Bourgi A . Learning curve on prostate fusion biopsies: key insights. World J Urol. 2025;43(1):215. 10.1007/s00345-025-05609-1 40192861

[17] Görtz M , Nyarangi‐Dix JN , Pursche L , Schütz V , Reimold P , Schwab C , et al. Impact of surgeon's experience in rigid versus elastic MRI/TRUS‐fusion biopsy to detect significant prostate cancer using targeted and systematic cores. Cancer. 2022;14(4):886. 10.3390/cancers14040886. PMC8870088 35205634

[18] Halstuch D , Baniel J , Lifshitz D , Sela S , Ber Y , Margel D . Characterizing the learning curve of MRI‐US fusion prostate biopsies. Prostate Cancer Prostatic Dis. 2019;22(4):546–551. 10.1038/s41391-019-0137-2 30842585

[19] Wallstrom J , Alterbeck M , Godtman RA , Bratt O , Jiborn T , Thimansson E . A comparison of magnetic resonance imaging assessment and biopsy outcomes with and without central review in two Swedish regional organized prostate cancer testing programs. Eur Urol Open Sci. 2025 Jul;77:32–38. 10.1016/j.euros.2025.05.008 40528941 PMC12173033

[20] Egevad L , Delahunt B , Berney DM , Bostwick DG , Cheville J , Comperat E , et al. Utility of pathology Imagebase for standardisation of prostate cancer grading. Histopathology. 2018 Jul;73(1):8–18. 10.1111/his.13471 29359484 PMC7838555

[21] Qin F , Liu Z , Ma J , Wu J , Shen Q , Liu Y , et al. Visibility of mpMRI region of interest on ultrasound during cognitive fusion targeted biopsy predicts prostate cancer detection: a prospective single‐center study. Abdom Radiol (NY). 2025 Jul;50(7):3305–3312. 10.1007/s00261-024-04750-6 39710761 PMC12181095

