# Supplementary material for: Comparison of software vs. cognitive‐based fusion‐targeted biopsies for prostate cancer diagnosis
Source: BJUI Compass. 2026 Jul 12;7(7):e70248. doi: 10.1002/bco2.70248 (PMC13357017; doi:10.1002/bco2.70248)
Supplement: Supplementary file 1 — Table S1. Target trial specification. STBx = Software‐based fusion‐targeted biopsies, CogTBx = cognitive fusion‐targeted biopsies. Figure S1. Centre biopsy technique preference, in centres that contributed to the main analysis, among all men who underwent software‐based fusion‐targeted biopsies (STBx) and cognitive fusion‐targeted biopsies (CogTBx) at these centres in 2020–2024. Figure S2. Number of men, biopsy technique and proportion with positive outcome according to centre in the main analysis. Centres were sorted according to the total number of men. STBx = Software‐based fusion‐targeted biopsies, CogTBx = cognitive fusion‐targeted biopsies. Figure S3. Absolute risk difference in the main analysis. Software‐based fusion‐targeted biopsies (STBx) versus cognitive fusion‐targeted biopsies (CogTBx). AR = absolute risk difference, CI = confidence interval. Figure S4. Relative risk and risk difference in the sensitivity analyses. Software‐based fusion‐targeted biopsies (STBx) versus cognitive fusion‐targeted biopsies (CogTBx). RR = relative risk. RD = absolute risk difference, CI = confidence interval. Figure S5. Distribution of inverse probability of treatment weights in the main analysis. STBx = Software‐based fusion‐targeted biopsies; CogTBx = cognitive fusion‐targeted biopsies. [file BCO2-7-e70248-s002.docx]

**Supplementary materials to**

Software vs. cognitive-based fusion-targeted biopsies for prostate cancer diagnosis: A target trial emulation

Table S1. Target trial specification.

| EF/TTF Attributes | Target trial | Emulation of the target trial using observed biopsy technique as exposure | Assumptions |
| --- | --- | --- | --- |
| Target population/ Eligibility criteria | Age 30-85 | Age 30-85 | None |
|  | PSA <20 | PSA <20 |  |
|  | MRI with PI-RADS 3-5 | MRI with PI-RADS 3-5 |  |
|  | No prior prostate cancer, not been biopsied the last 2 years, no use of finasteride or dutasteride the last 2 years, known prostate volume, low use of transperineal biopsies at centre | No prior prostate cancer, not been biopsied the last 2 years (KEB, TKE10/13/20/23), no use of finasteride or dutasteride the last 2 years, known prostate volume, low use (≤10%) of transperineal biopsies at centre during the corresponding calendar year |  |
| Treatment/Treatment strategies | **Intervention arm**: STBx.  **Control arm**: CogTBx. | **Intervention arm**: STBx (TKE20/23 and not TKE10/13).  **Control arm**: CogTBx (TKE10/13 and not TKE20/23). | Systematic biopsies may be performed in combination with targeted biopsies. Biopsies may be transrectal or transperineal. |
| Endpoint/Outcomes | Prostate cancer diagnosis (Any Gleason), prostate cancer diagnosis with Gleason score ≥ 3+4 and  prostate cancer diagnosis with Gleason score ≥ 4+3 | Prostate cancer diagnosis (Any Gleason), prostate cancer diagnosis with Gleason score ≥ 3+4 and  prostate cancer diagnosis with Gleason score ≥ 4+3 | None |
| Intercurrent events (IE) | Another biopsy within 30 days after the biopsy before any prostate cancer diagnosis | Another biopsy (KEB, TKE10/13/20/23) within 30 days after the biopsy before any prostate cancer diagnosis |  |
| Estimand | As treated (“*while on treatement*”) | Exposure: Biopsy with the observed technique | Prostate cancer diagnosis registered within 30 days from date of biopsy. Any additional biopsies in this 30-day period implies that the current biopsy did not lead to a prostate cancer diagnosis. |
|  | Average treatment effect (ATE) in the overlap population | Average treatment effect in weighted study population using overlap weights |  |
| Population-level summary/analysis plan | RR (95% CI) and AR (95% CI) | RR (95% CI) and AR (95% CI) |  |
| Assignment procedures | Randomisation to biopsy technique | Randomization is emulated by inverse probability of treatment weighting | Requires all confounders to be measured |
| Start/end follow-up | Start at the date of randomisation.  End at the date of biopsy. | Start at the date of biopsy.  End at 30 days after the date of biopsy. | The time between randomisation and intervention may introduce an immortal time bias in the emulation. This is ignored. |

EF, estimand framework; TTF, target trial emulation framework.

Figure S1. Centre biopsy technique preference, in centres that contributed to the main analysis, among all men that underwent software-based fusion-targeted biopsies (STBx) and cognitive fusion-targeted biopsies (CogTBx) at these centres in 2020-2024.


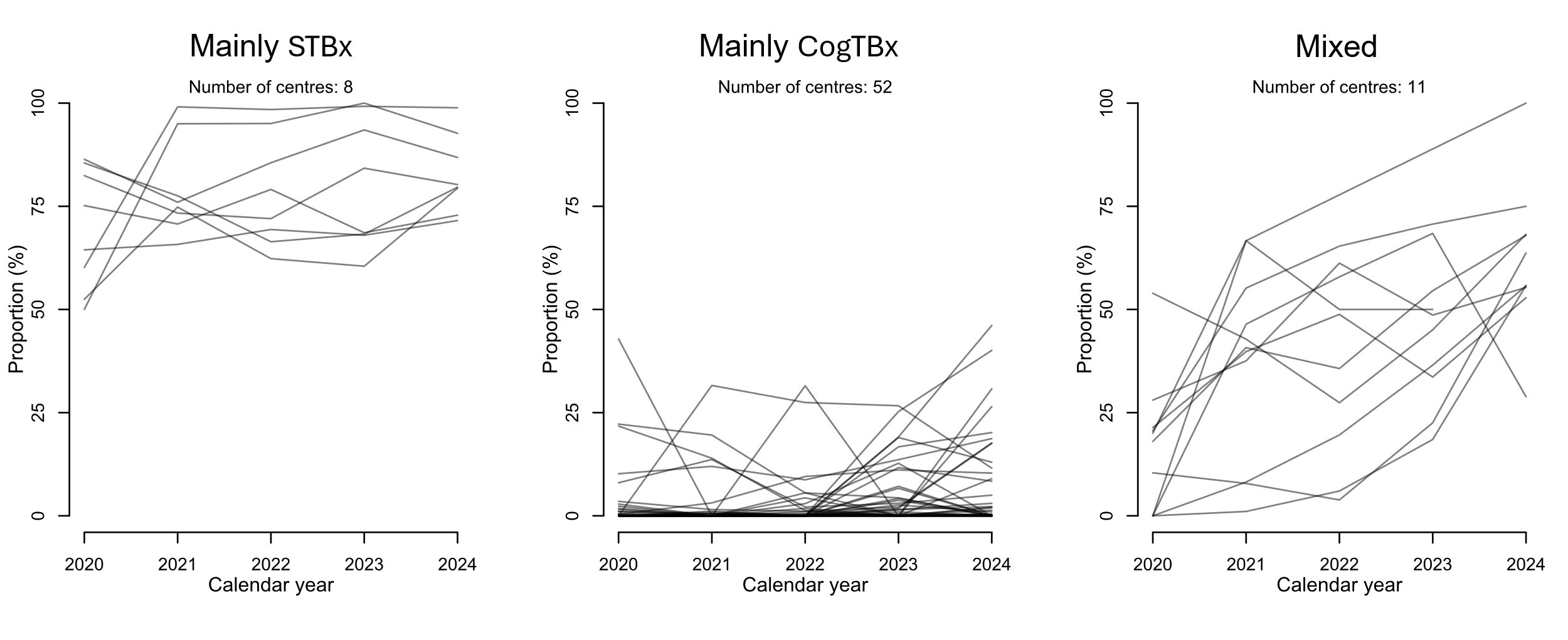


**Figure S2.** Number of men, biopsy technique and proportion with positive outcome according to centre in the main analysis. Centres were sorted according to the total number of men. STBx = Software-based fusion-targeted biopsies, CogTBx = cognitive fusion-targeted biopsies.


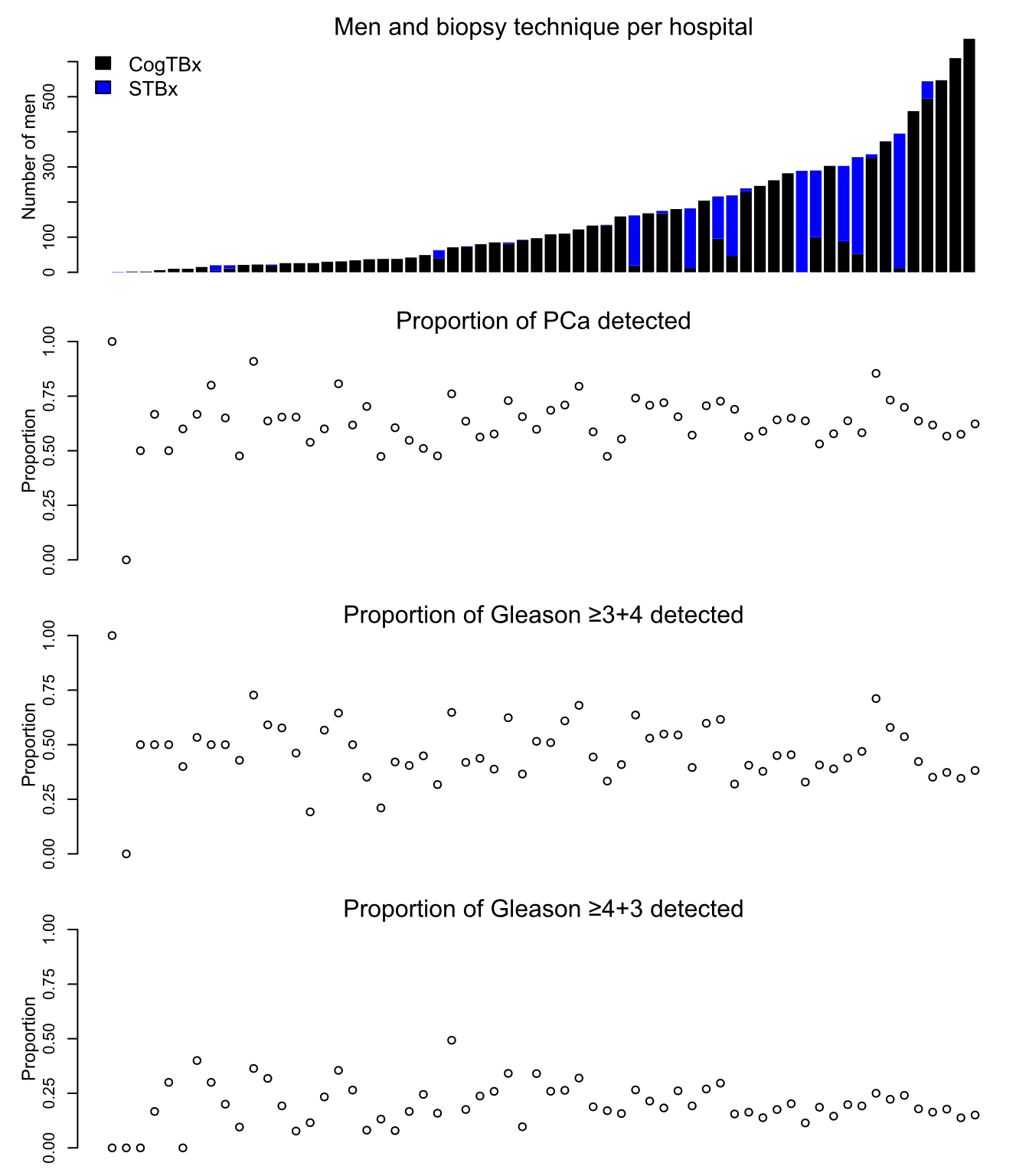


Figure S3. Absolute risk difference in the main analysis. Software-based fusion-targeted biopsies (STBx) versus cognitive fusion-targeted biopsies (CogTBx). AR=absolute risk difference, CI=confidence interval.


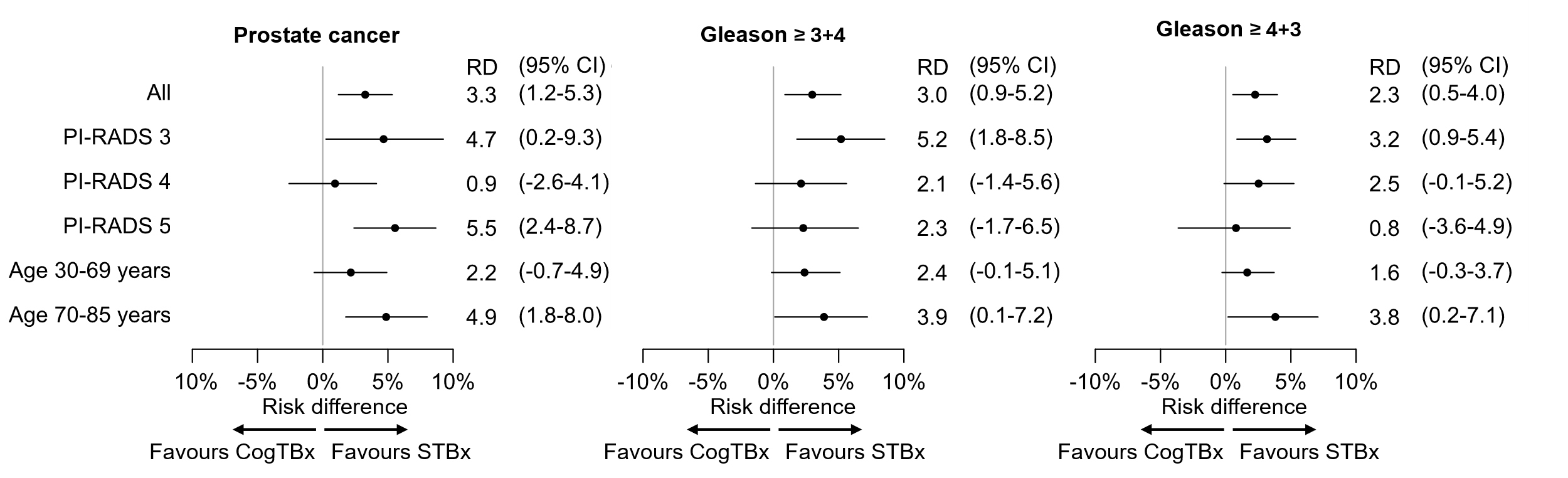


Figure S4. Relative risk and risk difference in the sensitivity analyses. Software-based fusion-targeted biopsies (STBx) versus cognitive fusion-targeted biopsies (CogTBx). RR=relative risk. RD=absolute risk difference, CI=confidence interval.


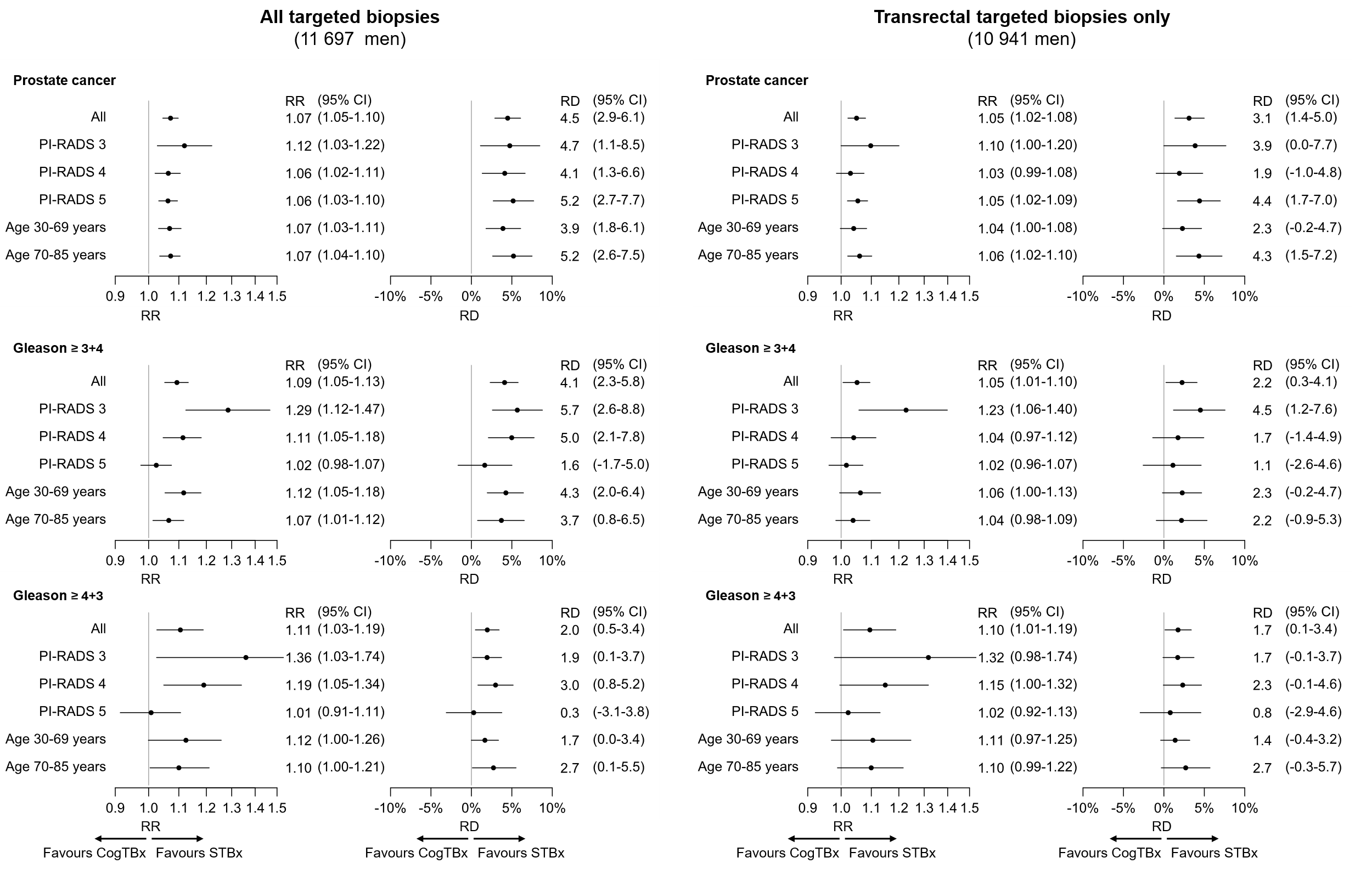


Figure S5. Distribution of inverse probability of treatment weights in the main analysis. STBx = Software-based fusion-targeted biopsies; CogTBx = cognitive fusion-targeted biopsies.
